# The Efficacy of Internet-Based Mindfulness Training and Cognitive-Behavioral Training With Telephone Support in the Enhancement of Mental Health Among College Students and Young Working Adults: Randomized Controlled Trial

**DOI:** 10.2196/jmir.6737

**Published:** 2017-03-22

**Authors:** Winnie WS Mak, Floria HN Chio, Amy TY Chan, Wacy WS Lui, Ellery KY Wu

**Affiliations:** ^1^ Department of Psychology The Chinese University of Hong Kong Hong Kong China (Hong Kong); ^2^ Center for Personal Growth and Crisis Intervention of the Corporate Clinical Psychology Services Hospital Authority Hong Kong China (Hong Kong)

**Keywords:** mental health promotion, Internet-based interventions, mindfulness-based training, cognitive-behavioral training, randomized controlled trial

## Abstract

**Background:**

College students and working adults are particularly vulnerable to stress and other mental health problems, and mental health promotion and prevention are needed to promote their mental health. In recent decades, mindfulness-based training has demonstrated to be efficacious in treating physical and psychological conditions.

**Objective:**

The aim of our study was to examine the efficacy of an Internet-based mindfulness training program (iMIND) in comparison with the well-established Internet-based cognitive-behavioral training program (iCBT) in promoting mental health among college students and young working adults.

**Methods:**

This study was a 2-arm, unblinded, randomized controlled trial comparing iMIND with iCBT. Participants were recruited online and offline via mass emails, advertisements in newspapers and magazines, announcement and leaflets in primary care clinics, and social networking sites. Eligible participants were randomized into either the iMIND (n=604) or the iCBT (n=651) condition. Participants received 8 Web-based sessions with information and exercises related to mindfulness or cognitive-behavioral principles. Telephone or email support was provided by trained first tier supporters who were supervised by the study’s research team. Primary outcomes included mental and physical health-related measures, which were self-assessed online at preprogram, postprogram, and 3-month follow-up.

**Results:**

Among the 1255 study participants, 213 and 127 completed the post- and 3-month follow-up assessment, respectively. Missing data were treated using restricted maximum likelihood estimation. Both iMIND (n=604) and iCBT (n=651) were efficacious in improving mental health, psychological distress, life satisfaction, sleep disturbance, and energy level.

**Conclusions:**

Both Internet-based mental health programs showed potential in improving the mental health from pre- to postassessment, and such improvement was sustained at the 3-month follow-up. The high attrition rate in this study suggests the need for refinement in future technology-based psychological programs. Mental health professionals need to team up with experts in information technology to increase personalization of Web-based interventions to enhance adherence.

**Trial Registration:**

Chinese Clinical Trial Registry (ChiCTR): ChiCTR-TRC-12002623; https://www2.ccrb.cuhk.edu.hk/ registry/public/191 (Archived by WebCite at http://www.webcitation.org/6kxt8DjM4).

## Introduction

According to the World Health Organization (WHO) [1], mental health is an essential part of health that contributes to the overall well-being of every individual. However, approximately 450 million people suffer from mental health problems worldwide [2]. Working adults are particularly vulnerable in Hong Kong. For instance, a survey found that 25% of the 1031 employees interviewed reported feeling down, depressed, or hopeless in the previous month, and 90% of them reported needing more mental health support at work [3]. Another survey conducted among 1207 employees also showed that 82.5% and 27.6 % of the employees suffered from stress and depression because of work, respectively [4]. Work stress can be detrimental to mental health and it is associated with the onset of depression and anxiety among the young working adults [5]. The Hong Kong Mental Morbidity Survey (HKMMS), which was the first territory-wide epidemiological study in Hong Kong, recruited 5719 Chinese participants in the general population of Hong Kong and found that 13.3% of adults have common mental disorder, with individuals aged 26-35 years having significantly higher weighted prevalence (16.5%) than the general adult population [6]. In view of their vulnerability to mental health problems, prevention programs are urgently needed to promote their mental health. As the WHO has suggested, mental health is not the mere absence of disease or infirmity but it also includes positive functioning and state of mind [1]. It is therefore important to provide a prevention program that does not focus only on the reduction of mental health problems or psychological distress but also promotes their positive functioning and state of mind.

In addition to working adults, emerging adults such as college students also experience high levels of stress. They are in the midst of identity exploration [7] and the instability involved in their transitional stage of becoming full-fledged adults may expose them to higher risks of psychological disturbance. Based on a survey conducted in 2006 covering 10 tertiary education institutions in Hong Kong, 21%, 41%, and 27 % of the 7915 first-year students reported moderate severity or above on depression, anxiety, and stress symptoms, respectively [8]. Another study also found high prevalence of depressive symptoms among college students, with 43.9% of the students reporting a score of 16 or above on the Center for Epidemiologic Studies Depression Scale, which is suggestive of depressive symptoms [9]. Thus, prevention and mental health promotion are also needed among college students.

Besides mental health, according to the WHO, physical health is also one of the components that is intimately related to mental health [1]. In Hong Kong, it has been found that pain and sleep disturbance are prevalent, with 80.3% of the 1051 Hong Kong Chinese adults who were interviewed indicating some pain over the past year [10] and 68.6% of the 529 Hong Kong college students interviewed reporting symptoms of insomnia [11]. Furthermore, pain, fatigue, and sleep disturbance are highly interconnected and the presence of all 3 physical problems is associated with poorer physical and mental health [12]. Since mental and physical health are highly interdependent [1], a mental health promotion program can potentially have benefits on physical health as well.

Although effective treatments are available, it is noted that two-thirds of people who suffered from mental disorder did not seek help due to the stigma in seeking mental health services [13]. In the HKMMS, only 26% of those with common mental disorders have sought mental health services in the past year, with only 3.9% having sought help from a psychologist [6]. Internet-based interventions provide an alternative to face-to-face therapy in enhancing mental health for people who may not seek help due to stigma or other reasons. It is anonymous, self-paced, and easily accessible. The high scalability and penetration of Internet-based interventions also offers advantages over face-to-face treatment.

Increasing evidence has shown the efficacy of Internet-based interventions in the treatment of anxiety and depression, as well as the promotion of mental health in the general public. Meta-analysis found the efficacy of Internet-based cognitive behavioral intervention in the treatment of anxiety and depression [14,15]. In addition to cognitive behavioral therapy, mindfulness-based interventions provide another means in enhancing mental and physical health. Mindfulness is a nonjudgmental awareness of the present moment with curiosity and openness. [16,17]. Among its many training programs, mindfulness-based stress reduction (MBSR) and mindfulness-based cognitive therapy (MBCT) are the most widely applied and are found to be efficacious in the treatment of depression and anxiety, as well as improving the physical and mental health conditions in the clinical and nonclinical populations [18-23]. Meta-analyses have also showed that mindfulness-based training is efficacious in the reduction of stress and anxiety among working adults and college students in clinical and community settings [24,25].

Although the face-to-face mindfulness-based interventions are efficacious, few have tested the efficacy when delivered through the Web. Two previous feasibility and pilot studies showed preliminary evidence of Internet-based mindfulness programs in improving stress in nonclinical population [26,27] and another randomized controlled trial showed the efficacy of Internet-based mindfulness training in enhancing quality of life among people in the clinical population [28]. In Hong Kong, one study showed that an 8-week Internet-based mindfulness training was efficacious in enhancing mental well-being among college students at postprogram and 3-month follow-up compared with the waitlist control [29]. Taken together, the preliminary evidence supported the feasibility of Internet-based mindfulness training in promoting mental health.

Although much work has been done on mindfulness training and Internet-based cognitive behavioral training, few have tested these Internet-based interventions in Asia. Also, the efficacy of Internet-based mindfulness training in promoting mental health is at its early stage. With the risks and prevalence of depression and anxiety observed among the college students and working adults, this study aimed to test the efficacy of an Internet-based mindfulness training for the prevention and promotion of their physical and mental health, compared with the well-established Internet-based cognitive behavioral training in a randomized controlled trial. We hypothesized that both training could enhance the physical and mental health at postprogram and 3-month follow-up.

## Methods

### Trial Design

This study was a 2-arm, randomized, open-label, parallel positive-control trial involving two Internet-based interventions: a mindfulness training program named iMIND versus a cognitive-behavioral training program named iCBT. Clinical ethics approval was obtained from the principal investigator’s institution (Joint Chinese University of Hong Kong–New Territories East Cluster Clinical Research Ethics Committee) as well as from the Hospital Authority Kowloon Central or East Cluster and the Department of Health of Hong Kong.

### Participants

The study targeted college students and young working adults and recruitment was done through (1) sending mass emails to students, teachers, and staff at different universities in Hong Kong; (2) distributing announcements to the staff of the Hospital Authority; (3) placing leaflets and posters in civil servant primary care clinics under Hong Kong Department of Health; and (4) posting advertisements in local libraries, newspapers, magazines, and social networking site Facebook.

Individuals who were interested in participating in the study visited our website where they were screened by completing Web-based questionnaires on mental health and demographics. Inclusion criteria included (1) age 18 years or above, (2) ability to read and understand Chinese, (3) computer literacy, and (4) consistent access to the Internet. Exclusion criteria included (1) an indication of suicidality by a score of 1-4 (out of 6) in item 16, 21, or 28 of the Mental Health Inventory (MHI) [30]; (2) currently receiving professional mental health services; and (3) currently taking psychotropic medication. If participants indicated suicidality in the screening questionnaire, they were to be given a list of resources and hotline on mental health services in the community.

Eligible individuals were given detailed information about the study aims, length of the program, participant involvement, and the assignment of intervention through randomization. They were also informed that the study was conducted by the Department of Psychology at the Chinese University of Hong Kong. Participants provided informed consent by clicking the “I agree” button at the bottom of the study description page. From there, participants received an activation link via email and were then randomly assigned to 1 of the 2 conditions by computer-generated numbers. The pre-, post-, and follow-up assessments were completed by the eligible participants on the Web, instead of through supporters, so that the assessment could be free from assessors’ biases from knowing the participants’ assigned conditions. Individuals who did not meet the eligibility criteria received an on-screen message and email with a thank you note and a list of resources on mental health services in the community.

### Interventions

iMIND and iCBT were administered via the Internet on 2 separate Web pages that were in the Chinese language. Functional tests were conducted before the release of the website. Each program consisted of 8 30- to 45-minute sessions. Both programs lasted for 8 weeks. The delivery format of iMIND involved didactic readings (eg, nature of human suffering according to the Buddhist perspective), experiential learning (eg, guided meditation), and daily life applications (eg, developing awareness on how letting go of one’s attachment could lead to inner peace). To enhance the user experiences, we made improvement on iMIND based on its predecessor [29] by making the content more interactive (eg, weekly well-being tracking, built-in multimedia within each lesson, dynamic content display) and more aesthetically appealing (eg, color coordinated and theme-consistent graphics with easy-to-use navigation). Recently, scholars have started to raise concerns about contemporary mindfulness teachings for their over-simplification and deviation from its traditional Buddhist root [31-33]. In response to this, our current iMIND program incorporated core notions in traditional Buddhism including discernment, compassion, impermanence, interdependence of all beings, and nonattachment [34]. By contextualizing our mindfulness training within the traditional Buddhist foundation, the training program aimed to facilitate participants to develop their own rationale behind practices. Such intentions would set the foundation for continuous and regular practices, and could potentially affect practice outcomes [35,36].

The content of iCBT was organized based on MacDonald and O’Hara’s 10 elements of mental health [37], with mental health promotion resources from the WHO and government reports from the United Kingdom and Australia. At the end of each session, participants were provided with homework assignments to practice what was learned and apply the skills in their daily lives. In the iMIND program, videos of stretching and audios of body scan and sitting meditation were provided to the participants to guide them through their exercises. In the iCBT program, worksheets including mood diary, cognitive restructuring, and healthy lifestyle plan were provided for participants to record their responses. All contents were developed by the research team members who were clinical psychologists and mindfulness practitioners. The iMIND and iCBT content was turned into the Web page by eLearningPro Limited. A brief overview of the session content is shown in Table 1. No further revision on the content and the Web page was made after the trial was launched. Screenshots of how the interventions appeared in the Web page are shown in Multimedia Appendix 1.

**Table 1:** Overview of session content.

Session	Content (iMIND^a^)	Content (iCBT^b^)
1	Introduction on mindfulness	Introduction on mental health
2	Observing thoughts, feelings, and sensations as they are	Stress, body reactions, and emotion regulations
3	Mindful attitudes and nature of suffering	Cognitive distortions and strategies to cope with stress
4	Being in the present moment	Emotion regulation
5	Letting go in times of difficulties	Resilience in times of adversities
6	Ways to stay mindful	Ways to increase self-esteem
7	Mindful communications	Effective communication skills
8	Review and applications	Review and applications

^a^iMIND: Internet-based mindfulness training program.

^b^iCBT: Internet-based cognitive-behavioral training program.

Previous research has shown that (1) guided self-help has higher completion rates than unguided self-help [38], (2) programs with weekly telephone reminders are more efficacious than those without [39], and (3) technician-assisted telephone or email support for Internet-based interventions is as efficacious as clinician-assisted telephone or email support [40,41]. Given these findings, for the duration of the 8-session program in both conditions, trained first tier supporters contacted each participant weekly via telephone and email to (1) acknowledge their time spent on the program, (2) ensure their understanding of course-related instructions, (3) encourage them to continue participating, and (4) provide guidelines for homework activities. Scripted guidelines and training were provided to the supporters. Participants were instructed to call and/or email our research assistant for clarification in case of questions or problems during the course of the intervention. When a participant fell below a score of 13 or answered 0 or 1 on any of the items on the Well-Being Index (WBI) [42], first tier supporters would refer them to second tier supporters (who were clinical psychologists) to evaluate their mental health status, address their questions, and make referrals for more intensive treatments as needed. Each participant was monitored through weekly self-report measure (ie, the WBI) as well as their first and second tier supporters. The first tier supporters also contacted the participants in both conditions once a month after the end of the program to maintain contact and interest in completing postprogram evaluations. The CONSORT e-health checklist is shown in Multimedia Appendix 2.

### Measures

#### Baseline Measures

At baseline, participants provided demographic and background information including age, gender, education level, income, marital status, religion, and previous experience with systematic mindfulness training (ie, mindfulness-based stress reduction therapy, MBSR, or mindfulness-based cognitive therapy, MBCT), regular meditation practices, cognitive-behavioral training, and yoga. To assess the route of participation, participants also indicated how they learned about this study.

#### Mental Health Measures

##### Mental Well-Being

The WHO 5-item WBI [43] was used to measure overall mental well-being. Each item was rated on a 6-point Likert scale from 0 (never) to 5 (all of the time). The scale has been used among the Chinese with an internal consistency of .90 [36]. In this study, the Cronbach alphas of the WBI were .92, .93, and .94 at baseline, postprogram, and 3-month follow-up, respectively.

##### Psychological Distress

The 18-item MHI was used to assess psychological distress [30]. Each item was rated on a 6-point Likert scale from 1 (all of the time) to 6 (none of the time). Previous research showed that the MHI’s internal consistency (Cronbach alphas) ranged from .81 to .91, with stability coefficients ranging from .60 to .76 over a 1-year interval [44]; its validity has also been supported among the Chinese. In this study, its Cronbach alphas were .93, .94, and .95, at baseline, postprogram, and 3-month follow-up, respectively.

##### Life Satisfaction

Life satisfaction was assessed by the 5-item Satisfaction with Life Scale (SWLS) [45]. Participants rated the extent to which they endorsed each item on a 6-point Likert scale from 1 (strongly disagree *)* to 6 (strongly agree *)*. Its reliability has been substantiated (eg, test-retest reliability of .84 over a 1-month interval) and its convergent validity has been demonstrated by its high correlations with other life satisfaction measures [46]. The scale has been used extensively among the Chinese and its validity has also been supported among Hong Kong university students [47]. In this study, the Cronbach alphas of the SWLS were .91, .91, and .90 at baseline, postprogram, and 3-month follow-up, respectively.

#### Physical Health Measures

##### Energy

Average level of energy was measured by the visual analogue scale (VAS) [48]. Participants rated their average daily energy level on a 100mm long line from 0 (no energy) to 100 (a lot of energy). Its validity is supported by its usage in measuring energy or fatigue level among patients with diagnoses of fatigue-related medical conditions [49].

##### Sleep Disturbance

The 4-item sleep disturbance subscale of the Medical Outcomes Study (MOS) Sleep Scale [50] was used to assess how well participants slept without tapping into other sleep-related medical conditions. Three items related to sleep disturbance were rated on a 6-point Likert scale from 1 (all of the time) to 6 (none of the time) and 1 item related to the time needed to fall asleep was assessed on a 5-point Likert scale from 1 (less than 15 minutes) to 5 (more than 60 minutes). Scores were converted to an index that ranged from 0-100 with higher scores indicating a higher level of sleep disturbance. Research has demonstrated its acceptable level of internal consistency reliability (>.70) and its responsiveness to change [50]. It has also been validated among community adults and has been used among the Chinese population [51]. The Cronbach alphas of MOS sleep scale were .83, .70, and .70 at baseline, postprogram, and 3-month follow-up, respectively.

##### Pain

Average level of pain was measured by VAS [52]. Participants rated their average daily pain level on a 100mm long line from 0 (no pain) to 100 (very severe pain). It has been used in a variety of settings and is sensitive to treatment effects [53]. It has been shown to be reliable in pain assessment when compared with other subjective pain measuring methods [54]. It also showed good reliability and validity among Chinese adults [55].

### Usage and Satisfaction Measures

Usage is defined as the time (in minutes) spent in the previous week on browsing the website and practicing the assigned homework. Participants reported these figures at the beginning of every session. At the end of the 8-week program, attitude toward and satisfaction with the Internet-based interventions were assessed using the Chinese version of the 8-item Client Satisfaction Questionnaire (CSQ) [56]. Each item was rated on a 4-point Likert scale from 1 to 4 and response options differed for different items. The Cronbach alpha of CSQ was .91 in this study.

### Credibility and Expectancy

At baseline, participants completed the 6-item Credibility or Expectancy Questionnaire (CEQ) that aimed to examine if expectancies or perception of treatment credibility were related to outcomes. Five items were rated on a 9-point Likert scale from 1 (not at all) to 9 (very much) and 1 item was rated on an 11-point Likert scale ranging from 0 (0%) to 11 (100%). The CEQ comprises 2 factors: cognitively based credibility and affectively based expectancy. It was shown to have a total item correlation of .78 [57] and the scale has been used among Chinese patients [58]. Standardized scores were computed for the 2 subscales. In this study, the Cronbach alphas for credibility and expectancy were .82 and .84, respectively.

### Analysis

All analyses were conducted using SPSS version 20.0 (IBM Corp) . Linear mixed models were conducted to test if both conditions showed improvements in all outcomes over time. Compound symmetry covariance was used and missing data were treated using restricted maximum likelihood estimation. Model for each outcome variable consisted of the time effect, condition effect, and the interaction effect of time by condition. When the main effect of time was significant, follow-up analyses were conducted to compare the outcomes in postprogram and follow-up program with the preprogram, and results were adjusted with Bonferroni correction. *T* tests were also conducted to test for equivalence in treatment expectancy and satisfaction about course content across the 2 conditions.

## Results

### Recruitment and Participant Characteristics

**Figure 1:**
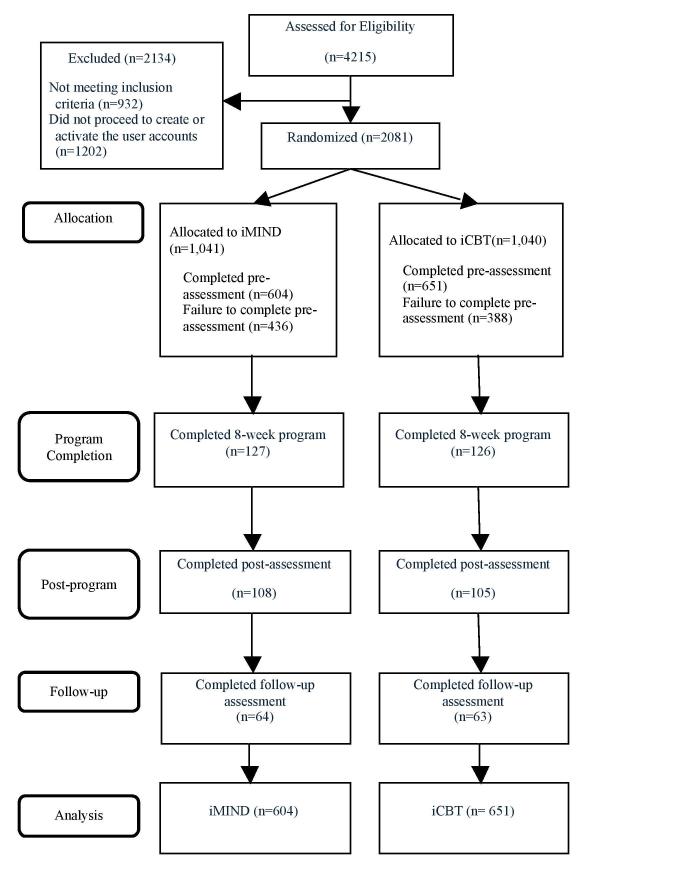
Flow diagram of this study.

Participants were recruited between July 2013 and March 2015. A total of 4215 registrants were screened for eligibility. Among those who registered, 932 (22.11%, 932/4215) registrants were deemed ineligible, 1202 (28.52%, 1202/4215) eligible registrants did not activate their accounts, whereas 2081 (49.37%, 2081/4215) eligible registrants proceeded with account activation followed by randomization. Our sample consisted of those who, after randomization, completed the presurvey and received course materials (N=1255). About one-fifth of the participants (n=253) completed the entire 8-session program, 16.97% (213/1255) completed the postprogram survey, and 10.12% (127/1255) completed the 3-month follow-up (see Figure 1 for the flow diagram). No adverse events were reported during the course of the study.

Participants learned about the study from a variety of avenues: work institutions or universities (36.65%, 460/1255), Facebook (28.21%, 354/1255), family or relatives or friends (21.27%, 267/1255), other means such as posters and leaflets (10.92%, 137/1255), and primary care clinics (2.95%, 37/1255). Tables 2 and 3 display the baseline characteristics of the participants in both conditions. Overall, participants had a mean age of 32.62 years (SD 12.54), were predominantly female (74.34%, 933/1255), with half of them being college graduates (52.51%, 659/1255). About one-thirds (34.6%, 434/1255) were college students and about half (51.07%, 641/1255) were working full-time (see Table 1). Both conditions reported similar treatment expectancy and credibility (*t*<0.57, *P*>.30). Findings showed that both conditions expressed similar CSQ usage satisfaction, *t*_211_ =−0.07, *P*=.94. In terms of utilization, iMIND condition (Mean 189.89, SD 501.00) spent more time browsing the course content than their iCBT counterparts (Mean 135.98, SD 347.08), *t*_1063.9_ =−2.20, *P*=.03). However, iCBT condition (Mean 240.63 minutes, SD 578.52) spent more time on homework assignment than iMIND condition (Mean 118.42, SD 401.42), *t*_1162.3_=4.37, *P*<.001.

To investigate the potential causes of attrition, we compared the baseline attributes between the attrition group (did not complete postprogram assessment; n=1042) and the retention group (n=213). No significant differences in their demographic characteristics were found, except for yoga experience. A slightly higher percentage of participants reported having had yoga experience in the retention group (28.6%) than in the attrition group (21.8%), χ^2^_1_ =4.5, *P*<.05. In terms of their psychological and stress profile, the attrition group was lower in mental well-being (WBI: Mean 2.04, SD 1.05; MHI: Mean 3.89, SD 0.84; SWLS: Mean 3.86, SD 1.40, *t*>2.32, *P*<.05), energy (Mean 53.11, SD 26.15; *t*_1253_=−2.01; *P*=.05), and treatment expectancy (credibility: Mean −0.05, SD 0.86; expectancy: Mean −0.03, SD 0.88) at preprogram than those who completed the postprogram assessment (WBI: Mean 2.25, SD 1.06; MHI: Mean 4.03, SD 0.80; SWLS: Mean 4.19, SD 1.35; energy: Mean 57.04, SD 25.65; credibility: Mean 0.23, SD 0.81; expectancy: Mean=0.16, SD=0.83).

**Table 2:** Baseline characteristics across conditions.

Characteristics		iCBT^a^ (n=651)	iMIND^b^ (n=604)
Age in years, mean (SD)		32.52 (12.41)	32.73 (12.68)
**Gender, n (%)**			
	Male	173 (26.6)	149 (24.7)
	Female	478 (73.4)	455 (75.3)
**Education, n (%)**			
	Primary or below	2 (0.3)	1 (0.2)
	Secondary	125 (19.2)	118 (19.5)
	Bachelor or diploma	346 (53.1)	313 (51.8)
	Master or above	178 (27.3)	172 (28.5)
**Employment, n (%)**			
	Student	226 (34.7)	208 (34.4)
	Full-time	331 (50.8)	310 (51.3)
	Part-time or freelance	29 (4.5)	28 (4.7)
	Others	65 (10)	58 (9.6)
**Religion, n (%)**			
	No religion	392 (60.2)	382 (63.1)
	Christianity	178 (27.3)	153(25.3)
	Catholicism	28 (4.3)	26 (4.3)
	Buddhism	41 (6.3)	34 (5.6)
	Others	12(1.9)	10 (1.7)
**Systematic mindfulness training, n (%)**			
	Yes	36 (5.5)	40 (6.6)
	No	615 (94.5)	564 (93.4)
**Regular meditation practices, n (%)**			
	Yes	60 (9.2)	57 (9.4)
	No	591 (90.8)	547 (90.6)
**Yoga experience, n (%)**			
	Yes	149 (22.9)	139 (23.0)
	No	502 (77.1)	465 (77.0)
**Cognitive behavioral, n (%) therapy experience, n (%)**			
	Yes	16 (2.5)	18 (3.0)
	No	635 (97.5)	586 (97.0)

^a^iCBT: Internet-based cognitive behavioral training program.

^b^iMIND: Internet-based mindfulness training program.

**Table 3:** Baseline characteristics across conditions.

Measures		iCBT^a^ (n=651), Mean (SD)	iMIND^b^ (n=604), Mean (SD)
Well-being index		2.02 (1.05)	2.14 (1.06)
Mental health inventory		3.90 (0.83)	3.93 (0.83)
Life satisfaction scale		3.90 (1.36)	3.94 (1.43)
Sleep disturbance		26.92 (20.24)	26.03 (20.69)
Pain		26.14 (25.75)	26.31 (25.62)
Energy		52.01 (26.41)	55.67 (25.64)
**Credibility or expectancy questionnaire**			
	Credibility	−0.02 (.86)	0.03 (0.86)
	Expectancy	0.01 (0.86)	−0.01 (0.88)	
			

^a^iCBT: Internet-based cognitive behavioral training program.

^b^iMIND: Internet-based mindfulness training program.

### Mental Health Measures

#### Well-Being Index

Results from the linear mixed model indicated a significant time effect (*P*<.001). Mental well-being significantly increased from baseline to postprogram (mean difference=−0.83, 95% CI −0.996 to −0.66, *P*<.001), and this increase was maintained at 3-month follow-up (mean difference=−0.73, 95% CI −0.94 to −0.51, *P*<.001) in both iMIND and iCBT. WBI was not significantly different between iMIND and iCBT (mean difference=−0.03, 95% CI −0.20 to 0.14, *P*=.75), and the 2 conditions did not differ in their improvements over time (*P*=.56)

#### Mental Health Inventory

The results indicated that there was a significant time effect (*P*<.001). It significantly increased from baseline to postprogram in both iMIND and iCBT (mean difference=−0.46, 95% CI −0.59 to −0.33, *P*<.001) and was maintained at 3-month follow-up (mean difference=−0.25, 95% CI −0.41 to −0.09, *P=*.001). MHI was not significantly different between iMIND and iCBT (mean difference=0.08, 95% CI −0.05 to 0.22, *P=*.22). The interaction effect of time x condition (*P*=.18) was also not significant, indicating that iMIND and iCBT showed similar improvement over time.

#### Life Satisfaction

The results indicated a significant time effect (*P*<.001), with life satisfaction significantly increased from baseline to postprogram in both iMIND and iCBT (mean difference=−0.77, 95% CI −0.96 to −0.59, *P*<.001), and this increase was maintained at 3-month follow-up (mean difference=−0.85, 95% CI −1.08 to −0.62, *P*<.001). The improvement was not significantly different between iMIND and iCBT (mean difference=−0.05, 95% CI −0.26 to 0.16, *P*=.62), and the time x condition interaction (*P*=.88), was not significant.

### Physical Health Measures

#### Energy

Results showed that energy improved over time (*P*<.001). The improvement was significant at postprogram, (mean difference=−12.60, 95% CI −16.54 to −8.67), *P*<.001), and was maintained at 3-month follow-up in both iMIND and iCBT, (mean difference=−13.42, 95% CI −18.37 to 8.47, *P*<.001). The effect did not differ between iMIND and iCBT (mean difference=−2.01, 95% CI −6.07 to 2.05, *P*=.33), and no significant interaction effect of time x condition (*P*=.67) was found.

#### Sleep Disturbance

Improvement was shown over time (*P*<.001). The improvement was significant at postprogram (mean difference=8.12, 95% CI 5.66-10.58, *P*<.001), and 3-month follow-up in both iMIND and iCBT (mean difference=7.46, 95% CI 4.39-10.53, *P*<.001). The improvement was not significantly different between iMIND and iCBT (mean difference=0.16, 95% CI −2.79 to 3.12, *P*<.001), and the interaction effect of time x condition (*P*=.91), was not significant.

#### Pain

Results showed that pain significantly improved over time (*P*=.01). The improvement was shown in postprogram (mean difference=3.95, 95% CI 0.27-7.63, *P=*.03). No significant effect was found for the condition effect (mean difference=−0.31, 95% CI −4.29 to 3.67, *P=*.88), and the time by condition effect (*P*=.58) were not significant. Tables 4 and 5 show a summary of the means, standard errors, effect sizes, and time effects of the outcome measures across conditions.

**Table 4:** Means and standard errors across conditions.

Measures^a^		iCBT^b^ (n=651)			iMIND^c^ (n=604)		
		Mean (SE^d^)			Mean (SE)		
		Pre	Post	Follow-up	Pre	Post	Follow-up
**Mental health measures**							
	WBI^e^	2.02 (0.04)	2.90 (0.10)	2.84 (0.12)	2.14 (0.04)	2.92 (0.10)	2.78 (0.12)
	MHI^f^	3.90 (0.03)	4.43 (0.07)	4.25 (0.09)	3.93 (0.03)	4.31 (0.08)	4.08 (0.09)
	SWLS^g^	3.90 (0.05)	4.69 (0.11)	4.71 (0.14)	3.94 (0.06)	4.69 (0.11)	4.82 (0.14)
**Physical health measures**							
	Energy	52.01 (0.99)	66.00 (2.26)	66.52 (2.87)	55.67 (1.03)	66.89 (2.29)	68.00 (2.89)
	Sleep disturbance	26.92 (0.77)	19.13 (1.51)	18.03 (1.85)	26.03 (0.81)	17.58 (1.53)	19.99 (1.88)
	Pain	26.14 (0.99)	21.04 (2.15)	23.12 (2.71)	26.31 (1.03)	23.50 (2.19)	21.43 (2.73)

^a^Significant time effects were shown for all measures (*P* s<.05). All post and follow-up scores were significantly improved compared with the prescores, except that pain did not show any improvement at follow-up compared with prescore. Interaction effect of time x condition were all nonsignificant, indicating that the improvements over time were similar across conditions.

^b^iCBT: Internet-based cognitive behavioral training program.

^c^iMIND: Internet-based mindfulness training program.

^d^SE: standard error.

^e^WBI: well-being index.

^f^MHI:Mental Health Inventory.

^g^SWLS: Satisfaction with Life Scale.

**Table 5:** Overall time effects and effect sizes across conditions.

Measures	Scales	iCBT^a^ (n=651)		iMIND^b^ (n=604)		Overall time effect			
		Cohen's d^c^		Cohen's d		Post versus pre mean difference (95% CI)	*P* value	Follow-up versus pre mean difference (95% CI)	*P* value
		Post versus pre	Follow-up versus pre	Post versus pre	Follow-up versus pre				
**Mental health measures**									
	WBI^d^	0.86	0.81	0.79	0.65	−0.83 (−0.996 to −0.66)	<.001	−0.73 (−0.94 to 0.51)	<.001
	MHI^e^	0.70	0.46	0.51	0.20	−0.46 (−0.59 to −0.33)	<.001	−0.25 (−0.41 to −0.09)	.001
	SWLS^f^	0.55	0.64	0.52	0.61	−0.77 (−0.96 to −0.59)	<.001	−0.85 (−1.08 to −0.62)	<.001
**Physical health measures**									
	Energy	0.56	0.58	0.45	0.49	−12.60 (16.54-8.67)	<.001	−13.42 (−18.37 to −8.47)	<.001
	Sleep disturbance	0.41	0.46	0.44	0.31	8.12 (5.66-10.58)	<.001	7.46 (4.39-10.53)	<.001
	Pain	0.21	0.12	0.11	0.20	3.95 (0.27-7.63)	.03	3.95 (−0.67 to 8.57)	.12

^a^iCBT: Internet-based cognitive behavioral training program.

^b^iMIND: Internet-based mindfulness training program.

^c^Cohen's d was computed from postprogram or 3-month follow-up score minus preprogram score divided by the pooled standard deviation.^d^WBI: Well-Being Index.^e^MHI: Mental Health Inventory.^f^SWLS: Satisfaction with Life Scale.

## Discussion

### Principal Findings

This study developed and evaluated the efficacy of the Internet-based mindfulness training in comparison with an Internet-based cognitive-behavioral training on college students and young working adults in Hong Kong. Results showed that the Internet-based mindfulness training was as efficacious as the widely supported Internet cognitive-behavioral training in improving mental well-being, psychological distress, life satisfaction, energy level, sleep disturbance, and pain at the end of the 8-week program. Furthermore, users’ perceived credibility, expectancy, and satisfaction of both programs were similar. The results are encouraging as both Internet-based programs received support for their utility, satisfaction, and efficacy in mental health promotion. Given the weight of mental illness disease burden in our communities, this study shows that Internet-based mindfulness and cognitive-behavioral training programs with minimal guided support can be a highly scalable and convenient way for prevention and promotion of mental and physical health among college students and young working adults

In Hong Kong, the majority of individuals who seek help for mental health issues do not receive psychiatric and clinical psychological services in primary and secondary care settings until their problems have become severe. In comparison with face-to-face interventions, Internet-based interventions are more easily accessible and affordable and have the potential to fulfill the need for mental health promotion and prevention in community settings. This study provided empirical support for the efficacy of Internet-based cognitive-behavioral and mindfulness training programs, which can be easily incorporated into existing service provision portfolios that promote mental health and reduce psychological distress among the college students and young working adult population in Hong Kong.

In terms of service management, these developed Internet-based interventions are highly sustainable. In Hong Kong, the number of mobile phone customers reached over 8 million in June 2016, and the amount of mobile data usage has been 10-folded from 2006 to 2016, demonstrating the rapid increase of mobile phone and mobile Internet usage [59]. With the high penetration of Internet-based programs and the increasing prevalence of mobile phone and tablet device utilization, Internet-based programs meet the public mental health goal of reaching the general public for mental health promotion and prevention under the stepped care model, especially if the current programs can be converted into mobile phone apps in the future. Although attrition may be high, given it is an easily accessible public health tool, if its dissemination in the population is wide, such Internet-based mental health promotional tools can still be an important augmentation to face-to-face interventions in filling the role of mental health promotion and illness prevention that is lacking in the current mental health services system in Hong Kong [60]. One size does not fit all; we need myriad approaches of varying dosage and penetration rates to reach a wider population in order to prevent the tremendous mental illness burden [61,62]. Thus, we believe Internet-based programs still have their merits by reaching out to many more individuals at a much shorter period of time. Even if only a tenth of the thousands improved over the course of the programs, it is still worthwhile to make this accessible to individuals who may not have access or do not prefer to have face-to-face interventions.

Future research should explore methods for enhancing adherence of Internet-based health solutions in order to harness the expanding proliferation of technology among the public. For instance, recent studies have begun to incorporate ecological momentary intervention components into Internet-based programs so that interventions can be directed to real-time events and be more personalized [63,64]. Emerging evidence has suggested efficacy of ecological momentary interventions in the enhancement of a variety of health behaviors [65]. To leverage the power of technology in the promotion of mental health, interventions that incorporate these methods may reduce attrition rate and further maximize the efficacy of the Internet-based programs.

Although improvements in outcomes were observed at postprogram, the improvements for pain were not maintained at 3-month follow-up. This could be the result of reduced practice or application of skills learned on the websites. In addition, this might also due to the low level of pain observed within this group of population. The floor effect might have limited the possibility in detecting improvement in pain at postprogram and 3-month follow-up.

The two Internet-based interventions in this study yielded similar results. Future studies can explore how individual differences may affect intervention benefits. It may be possible that cognitive styles can play a role in the receptivity of iMIND and iCBT and matching their styles with the treatment approach may maximize the outcome.

### Limitations

This study has several limitations. First, our target population was college students and young working adults. By nature, our sample is skewed toward those who were educated or were employed. As our programs were Internet-based, it is possible that they appealed to a selective group in the population who were more comfortable in accessing interventions over the Internet with their personal computers. They might have higher mental health literacy and be more willing to participate in Internet-based mental health programs. These biases in our sample limit the generalizability of our findings to all segments of the population (eg, less educated individuals, older adults). It is possible that the delivery of mental health materials over the Internet may only be appropriate for specific segments of the populations, rather than the entire population. Future studies should focus on how Internet-based interventions can cater to different segments of the populations through various adaptations.

Second, the attrition rate of our study is high. High attrition rate has been a perennial problem for Internet-based interventions. Similarly high attrition rates have been reported in other Internet-based mental health programs. For example, Christensen and colleagues [66] reported an attrition rate of 74% in their Internet-based cognitive behavioral therapy (CBT) program for depression. Another study reported an attrition rate of 98.8% in an Internet-based CBT for panic disorder [67]. A study based on the MoodGym had an attrition rate of 73.9% for trial participants and 99.2% for public registrants [68]. In a more recent systematic review of Internet-based interventions for anxiety and depression, the completion of protocol rates for depression sites ranged from 43% to 99% [69]. Another systematic review on Internet-based interventions for psychological disorders also found that there was an additional 0% to 18% of participants who would further dropout from post to follow-up assessment [70]. Moreover, during the inception of our project, mobile phones and tablets were not as omnipresent as they are today. Because of that, our Web-based modules were designed using Adobe Flash and thus only catered for desktop viewing, which may deter usage.

Third, we did not include a waitlist control group in this study. As this study aimed to compare Internet-based mindfulness training with a well-established Internet-based cognitive behavioral training, and previous study has found Internet-based mindfulness training to have significant improvements in mental health than waitlist control [29], we decided not to have a waitlist control in this study so as not to withhold intervention from our participants. Fourth, we did not ask whether our participants received any other psychological intervention during the study period. Thus, the findings may potentially be attributed to additional intervention that the participants have received. Finally, this study found that those who quit the programs scored lower on mental health measures, energy level, mindful awareness, and treatment expectancy at the outset. To ensure interventions are catered to those most in need, future studies should explore the reasons behind attrition and identify corresponding remedies. As suggested by existing research, utilization can potentially be promoted via built-in incentives, personalized feedback, and user collaboration [71].

### Conclusions

In sum, this study showed that both Internet-based mindfulness training and Internet-based cognitive-behavioral training were efficacious in improving mental and physical health indicators among college students and young working adults in a convenient fashion. To leverage the power of technology in reducing mental illness burden, it is paramount for mental health professionals to work in tandem with professionals in other disciplines (eg, designers, computer scientists) in creating user-friendly programs that enable seamless integration into users’ daily lives.

## Appendix

Multimedia Appendix 1 Screenshots of the iMIND and iCBT.

Multimedia Appendix 2 CONSORT EHEALTH checklist.

## Abbreviations

CBT: cognitive behavioral therapy
CEQ: Credibility or Expectancy Questionnaire
CSQ: Client Satisfaction Questionnaire
HKMMS: Hong Kong Mental Morbidity Survey
MBSR: mindfulness-based stress reduction
MBCT: mindfulness-based cognitive therapy
MHI: Mental Health Inventory
SWLS: Satisfaction with Life Scale
VAS: visual analogue scale
WBI: Well-Being Index
WHO: World Health Organization
