# Supplementary material for: The Efficacy of Internet-Based Mindfulness Training and Cognitive-Behavioral Training With Telephone Support in the Enhancement of Mental Health Among College Students and Young Working Adults: Randomized Controlled Trial
Source: J Med Internet Res. 2017 Mar 22;19(3):e84. doi: 10.2196/jmir.6737 (PMC5382258; doi:10.2196/jmir.6737)

Multimedia Appendix 1: Screenshots of the iCBT and iMIND

Sign in Page

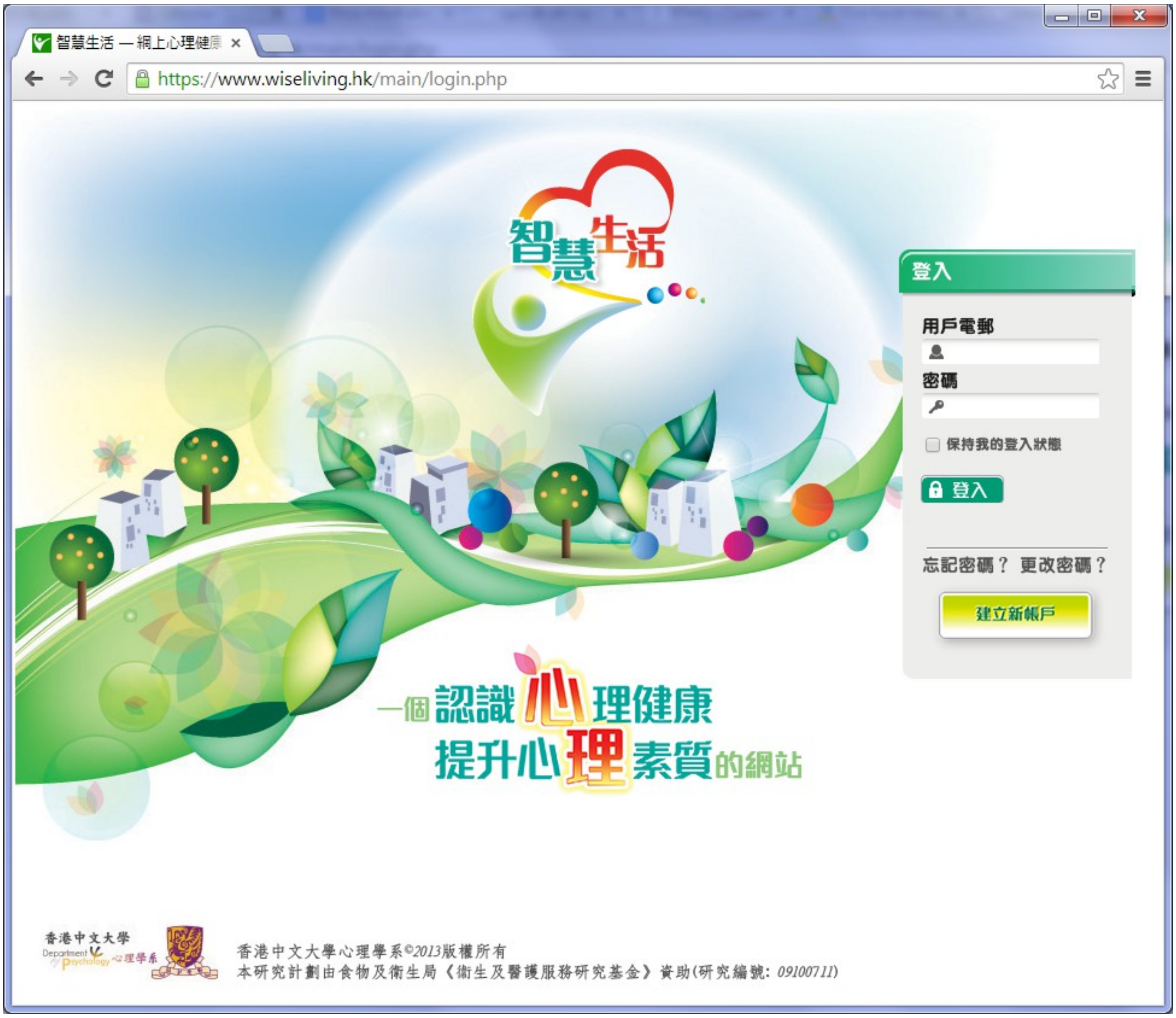
智慧生活 — 網上心理健康
https://www.wiseliving.hk/main/login.php
智慧生活
登入
用戶電郵
密碼
保持我的登入狀態
登入
忘記密碼？ 更改密碼？
建立新帳戶
一個認識心理健康
提升心理素質的網站
香港中文大學心理學系©2013版權所有
本研究計劃由食物及衛生局《衛生及醫護服務研究基金》資助(研究編號: 09100711)

Informed Consent

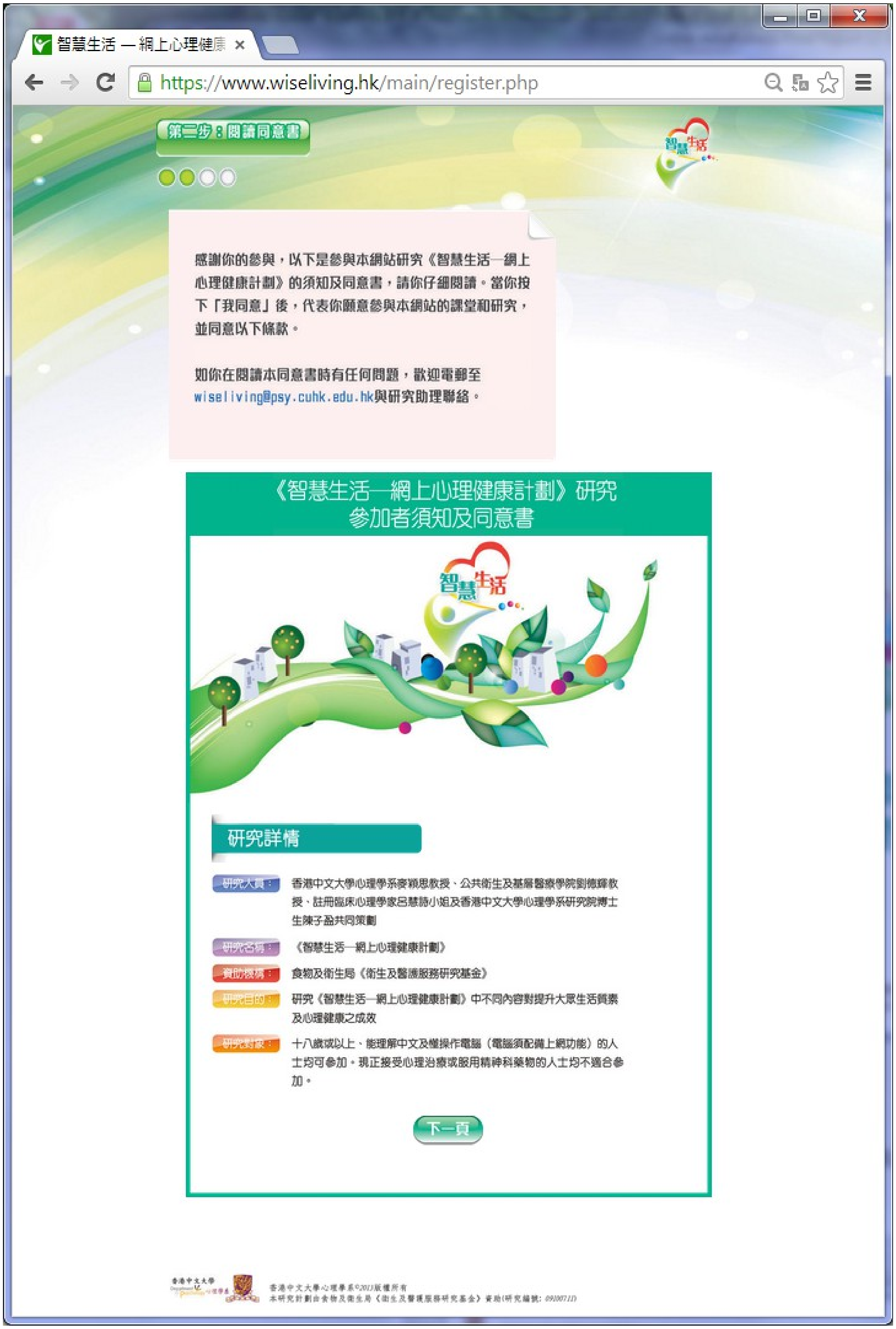

Informed Consent (continued)

《智慧生活─網上心理健康計劃》研究
參加者須知及同意書

研究詳情

研究程序：

剛才所填寫的問卷是為了篩選合資格的參加者，資料會絕對保密，並不會作進一步分析之用。合資格人士於填妥篩選用之問卷後，會進入《參加者須知及同意書》部分，參加者必須按「我同意」，以表示清楚了解研究內容及同意參加是次研究項目。

參加者會隨意被分派到兩個組別的其中一個。一組的參加者將會接受為期八個星期的心理教育課程，而另一組將會接受為期八個星期的靜觀生活課程。根據過往研究顯示，兩個組別均能對精神健康有所幫助。

參加者開設個人帳戶後，均需於第一節課前、第八節課後及完成課程後三個月於網上填寫問卷，以檢討計劃成效。問卷內容包括評估參加者的身心狀況、思考模式及生活滿意度。

每個帳戶的課堂進度及問卷紀錄都會被儲存在電腦系統內，因此請參加者避免與他人分享帳戶。使用本網站費用全免，歡迎有興趣人士自行開設獨立帳戶。

Informed Consent (continued)

在課堂進行的八星期裹，本計劃的工作人員，包括研究助理、臨床心理學家等會每星期致電參加者，了解參加者的狀況，而電腦系統也會透過電郵向參加者發出通知，因此系統要求參加者提供電話號碼和電郵等個人資料。為保障安全，這些資料將會被加密傳送，而且資料會被絕對保密。

如參加者對本課程有任何查詢，可透過電話或電郵諮詢研究助理，研究助理會將較深入的問題交由本研究團隊的臨床心理學家解答。假若參加者的每週心理健康分數低於八分及出現自殺念頭或有需要接受進一步的心理評估時，研究助理會把參加者轉介給研究團隊的臨床心理學家跟進，臨床心理學家會以電話形式跟參加者進行一次簡短的評估，並根據參加者的不同需要，作出合適的轉介服務。經臨床心理學家評估後，所有參加者（包括已接受轉介服務之人士）可繼續餘下的課程。首四百位完成八堂課程及課後評估的參加者，可獲得港幣五十元現金券（可供選擇的商店包括百佳超級市場、Starbucks及Haagen-Dazs雪糕店）。現金券須於2014年8月31日或之前到沙田中文大學心理系領取。所有完成跟進評估的參加者，可獲得個人報告乙份（寄至參加者的電子郵箱）。

研究性質： 研究會透過互聯網進行，參加者需於八個星期內，每星期花三十至四十五分鐘，登上網站，瀏覽指定資訊。課程期間，參加者亦需完成有關的功課或練習，以深化課程之學習。

參與是項研究涉及之風險： 根據現時的文獻顯示，本項研究將不會令參加者產生任何特別之不安，亦沒有任何潛在的危險。

根據香港法律規定（特別是第 486 章《個人資料（私隱）條例》），參加者享有或可享有確保你的個人資料保密的權利，例如在或為本研究中有關收集、監管、保留、管理、控制、使用（包括分析或比較）、轉進或轉出香港、不披露、清除和/ 或以任何方式處理或棄置的權利。如有任何問題，請你咨詢個人資料私隱專員或其職員（電話號碼：2827 2827），以瞭解妥善監控或監管你的個人資料保護之事宜，以確保你完整掌握和瞭解遵守規管個人資料私隱的法律之重要性。

下一頁

Table of Contents for iCBT

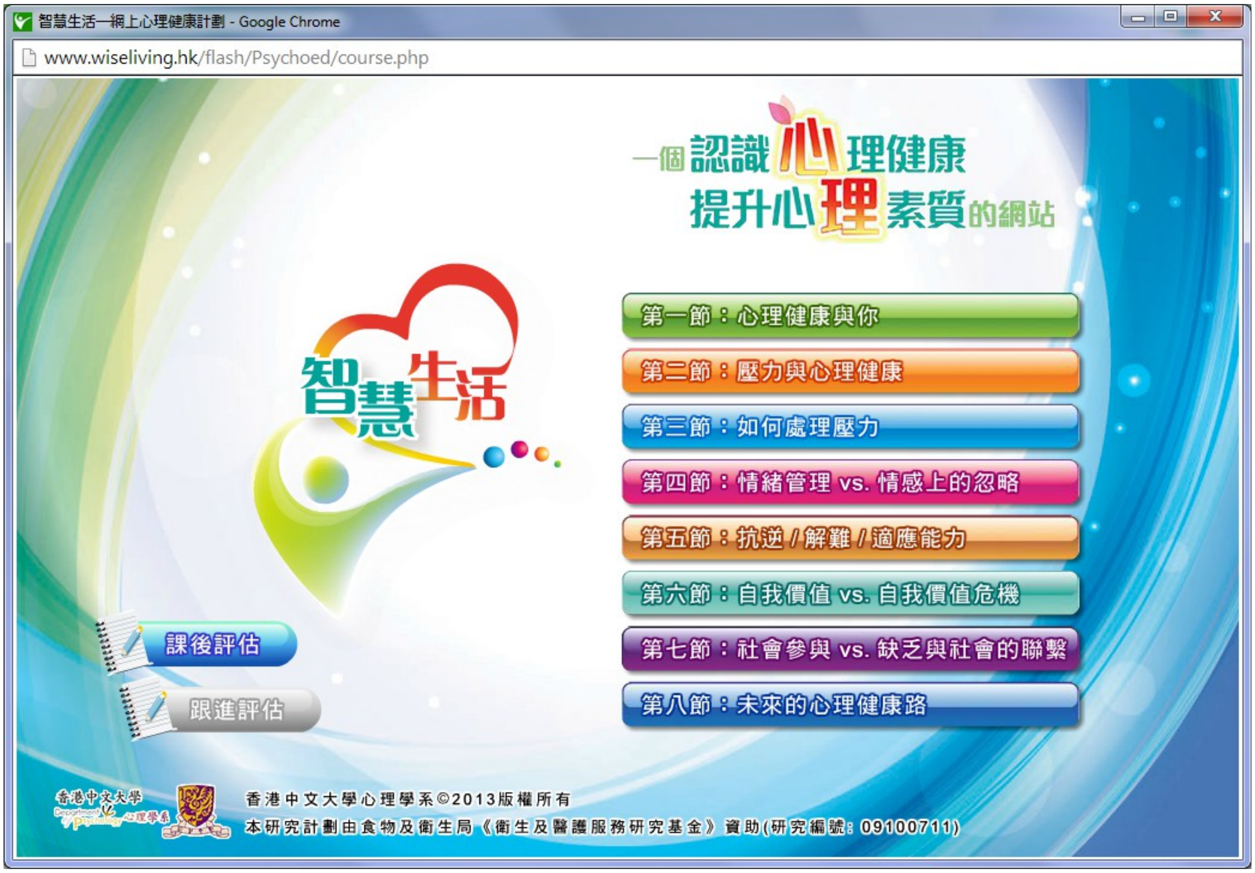

Sample Content from iCBT

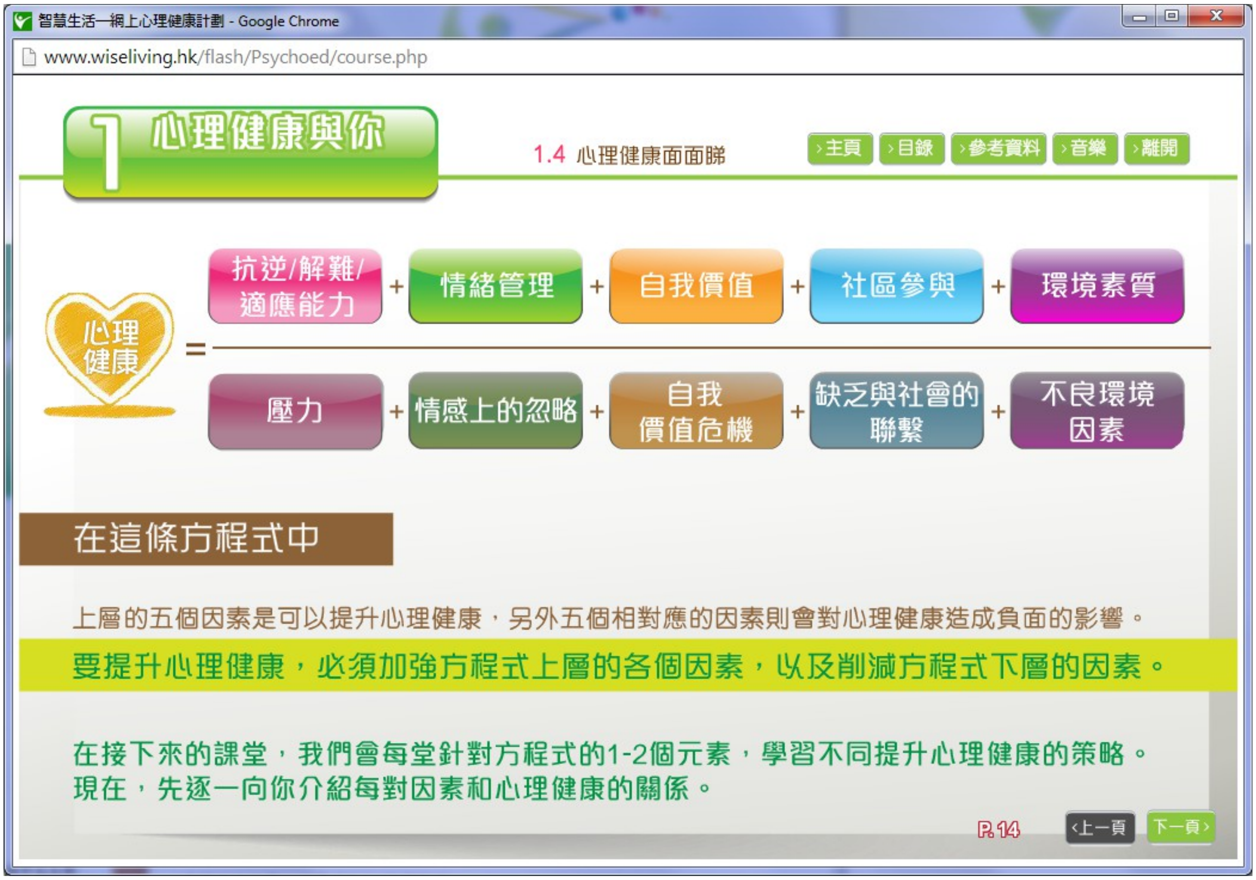

Table of Contents for iMIND

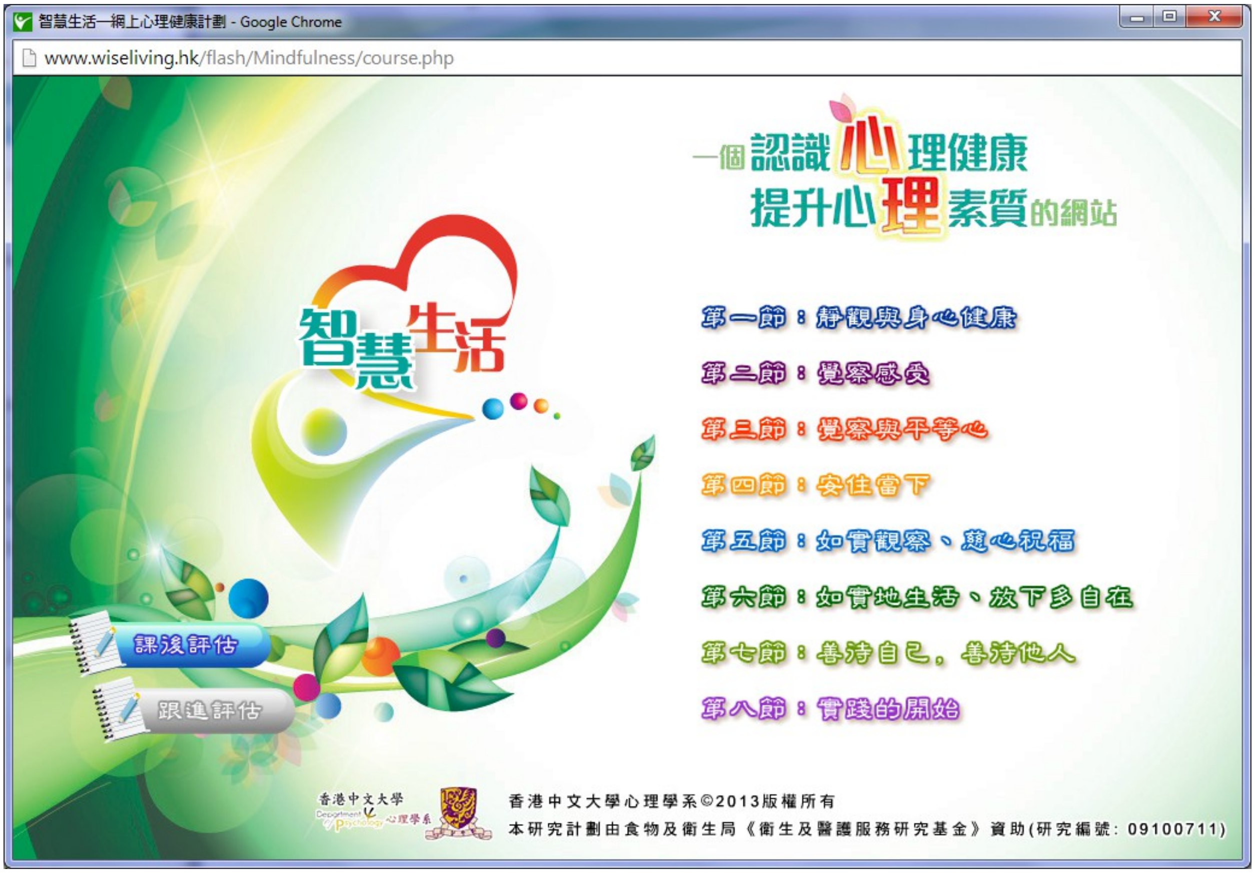

Sample content from iMIND

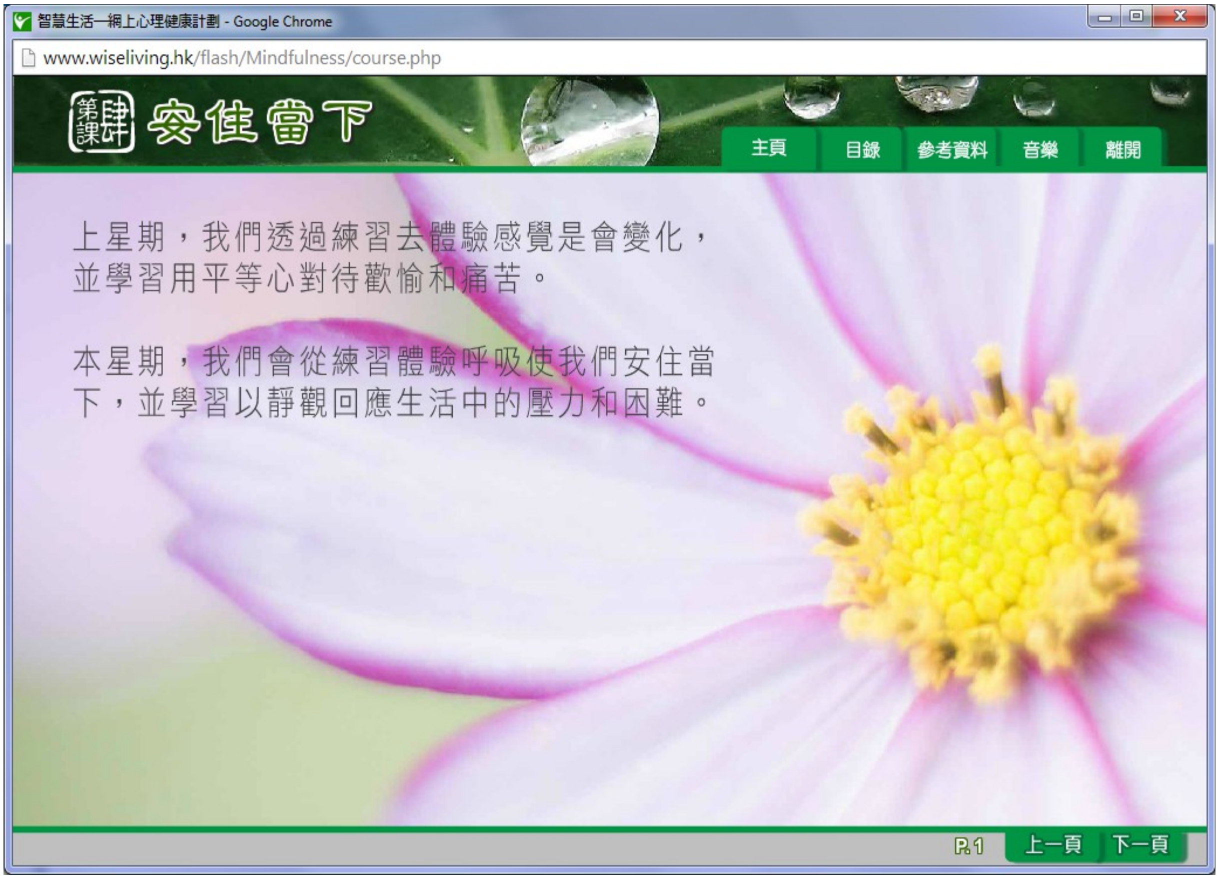

Supplement: Multimedia Appendix 1 [file jmir_v19i3e84_app1.pdf]
