# Supplementary material for: The Efficacy of Internet-Based Mindfulness Training and Cognitive-Behavioral Training With Telephone Support in the Enhancement of Mental Health Among College Students and Young Working Adults: Randomized Controlled Trial
Source: J Med Internet Res. 2017 Mar 22;19(3):e84. doi: 10.2196/jmir.6737 (PMC5382258; doi:10.2196/jmir.6737)
Supplement: Multimedia Appendix 2 [file jmir_v19i3e84_app2.pdf]

CONSORT-EHEALTH Checklist V1.6.2 Report

Date completed

10/3/2016

by

Winnie W. S. Mak

The efficacy of Internet-based mindfulness training and cognitive-behavioral training with telephone support in the enhancement of mental health among college students and young working adults: A randomized controlled trial

1a) Yes. The title included the word “Internet-based” to indicate the nature of the interventions.

1a-ii) Yes, the title included “with telephone support” to indicate the non-web-based component.

1a-iii) Yes, the title included “among college students and young working adults” to indicate the target group of the study.

1b) Yes. “Our aim is to examine the effectiveness of an Internet-based mindfulness training (iMIND) in comparison with the well-supported Internet-based cognitive-behavioral training (iCBT) in promoting mental health”

1b-ii) Yes. “Telephone and/or email support were provided by trained first tier supporters who were supervised by the study’s research assistants.”

1b-iii) Yes. “Participants were recruited online and offline via mass mail, announcement and leaflets in clinics, and social networking site.” “Primary outcomes included indicators of mental and physical well-being, which were self-assessed online at pre-program, post-program, and 3-month follow-up.

1b-iv) Yes. “Among the 1,255 study participants, 213 and 127 completed the post- and 3-month follow-up assessment, respectively… Both iMIND (n = 604) and iCBT (n = 651) were effective in improving mental well-being, psychological distress, life satisfaction, sleep disturbance, and energy level.”

1b-v) Yes. “Both Internet-based mental health programs showed potential in improving the well-being from pre- to post-assessment, and such improvement was sustained at 3-month follow-up.”

2a-i) Yes. “With the risks and prevalence of depression and anxiety observed among the college students and working adults, the present study aimed to test the effectiveness of an Internet-based mindfulness training in improving their well-being, compared with the well-established Internet-based cognitive behavioral training in a randomized controlled trial.”

2a-ii) Yes. “Although the face-to-face mindfulness-based interventions are effective, few have tested the effectiveness when delivered online. Two previous feasibility and pilot studies showed preliminary evidence of Internet-based mindfulness programs in improving stress in non-clinical population [23, 24] and another randomized controlled trial showed the effectiveness of Internet-based mindfulness training in enhancing quality of life among people in the clinical population [25]. Although much work has been done on mindfulness training and Internet-based cognitive behavioral training, few have tested these Internet-based interventions in Asia. Also, the effectiveness of Internet-based mindfulness training in promoting mental health is at its early stage.”
2b) Yes. “The present study aimed to test the effectiveness of an Internet-based mindfulness training in improving their well-being, compared with the well-established Internet-based cognitive behavioral training in a randomized controlled trial. We hypothesized that both training could enhance well-being at post-assessment and 3-month follow-up.”

3a) Yes. “This study was a two-arm, randomised, open-label, parallel positive-control trial involving two Internet-based interventions: a mindfulness training program named iMIND versus a cognitive-behavioral training program named iCBT.”
3b) No. There is no change on methods after trial commencement
3b-i) No. There was no system change after trial commenced.

4a) Yes. “Inclusion criteria included: (1) being 18 years of old or above, (2) being able to read and understand Chinese, (3) being computer literate, (4) having consistent access to the Internet. Exclusion criteria included (1) an indication of suicidality by a score of 1-4 (out of 6) in item 16, 21, or 28 of the Mental Health Inventory (MHI), (2) currently receiving professional mental health services, and (3) currently taking psychotropic medication.”
4a-i) Yes. “Inclusion criteria included: (1) being 18 years of old or above, (2) being able to read and understand Chinese, (3) being computer literate, (4) having consistent access to the Internet.”
4a-ii) Yes. The study targeted college students and young working adults and recruitment was done through (1) sending mass emails to students, teachers, and staff at different universities in Hong Kong, (2) distributing announcements to staff of the Hospital Authority, (3) placing leaflets and posters in civil servant primary care clinics under Hong Kong Department of Health, and (4) posting advertisements in local libraries, newspapers, magazines, and a social networking site (i.e., Facebook).

To prevent multiple identities, it has also been mentioned in the manuscripts that "participants received an activation link via email"
4a-iii) Yes. "Eligible individuals were given detailed information about the study aims, length of the program, participant involvement, and the assignment of intervention through randomization. They were also informed that the study was conducted by the Department of Psychology at The Chinese University of Hong Kong."
4b) Yes. "Participants filled in the pre- post-, and follow-up assessments online."
4b-i) Yes. "Participants filled in the pre- post-, and follow-up assessments online."
4b-ii) Yes. "They were also informed that the study was conducted by the Department of Psychology at The Chinese University of Hong Kong."

5) Yes. "All contents were developed by the research team members who were clinical psychologists and mindfulness practitioners." Conflict of interest: "The study was supported by the Health and Health Services Research Fund (Ref. No. 09100711). The first author of the study, Winnie Mak, is one of the developers of the content of the trials but do not own the source code of the website." Content was developed by the eLearningPro Limited.
5-ii) Yes. "The content of iMIND was based on MBSR and was identical to the previous randomized controlled trial on Internet-based mindfulness training conducted by the authors except that it was enhanced in terms of interactivity (e.g., more in-class exercises and audios) and aesthetics (e.g., more graphics and illustrations)."
5-iii) No major change was made after the website was launched.
5-iv) Yes. "Functional tests were conducted prior to the release of the website"
5-v) Yes, screenshots were provided in Multimedia Appendix 1.
5-vi) Since no funding support is available for the website to keep running, it is no long accessible but screenshots are available and were shown in the Appendix.
5-vii) The participant accessed the content with an URL for free.
5-viii) Yes. "Each programme consisted of eight 30- to 45-minute sessions. The content of iMIND was based on MBSR and was identical to the previous randomized controlled trial on Internet-based mindfulness training conducted by the authors [26] except that it was enhanced in terms of interactivity (e.g., more in-class exercises and audios) and aesthetics (e.g., more graphics and illustrations). The content of iCBT was organized based on MacDonald and O'Hara's ten elements of mental health [27] with mental health promotion resources from the WHO and government reports from the United Kingdom and Australia. At the end of each session, participants were provided

with homework assignments to practice what was learned and apply the skills in their daily lives. In the iMIND, videos of stretching and audios of body scan and sitting meditation were provided to the participants to guide them through their exercises. In the iCBT, worksheets including mood diary, cognitive structuring, and healthy lifestyle plan were provided for participants to record their responses. All contents were developed by the research team members who were clinical psychologists and mindfulness practitioners. Previous research has shown that (1) guided self-help has higher completion rates than unguided self-help [27], (2) programs with weekly telephone reminders are more effective than those without [28], and (3) technician-assisted telephone/email support for Internet-based interventions is as effective as clinician-assisted telephone/email support [29-30]. Given these findings, for the duration of the 8-session program in both conditions, trained first tier supporters contacted each participant weekly via telephone and/or email to: (1) acknowledge their time spent on the program, (2) ensure their understanding of course related instructions, (3) encourage them to continue participating, and (4) provide guidelines for home work activities. Scripted guidelines and training were provided to the supporters. Participants were instructed to call and/or email our research assistant for clarification in case of questions or problems during the course of the intervention"

5-ix) Yes. "Each programme consisted of eight 30- to 45-minute sessions." "At the end of each session, participants were provided with homework assignments to practice what was learned and apply the skills in their daily lives." Adherence to the practice duration was ad libitum.

5-x) Yes. "For the duration of the 8-session program in both conditions, trained first tier supporters contacted each participant weekly via telephone and/or email to: (1) acknowledge their time spent on the program, (2) ensure their understanding of course related instructions, (3) encourage them to continue participating, and (4) provide guidelines for home work activities. Scripted guidelines and training were provided to the supporters. Participants were instructed to call and/or email our research assistant for clarification in case of questions or problems during the course of the intervention. When a participant fell below a score of 13 or answered 0 or 1 on any of the items on the Well-Being Index [31], first tier supporters would refer them to second tier supporters (who were clinical psychologists) to evaluate their mental health status, address their questions, and/or make referrals for more intensive treatments as needed. Each participant was monitored through weekly self-report measures as well as their first and/or second tier supporters. The first tier supporters also called the participants in both conditions once a month after the end of the program to maintain contact and interest in completing post-program evaluations."

5-xi) Yes. “The first tier supporters also called the participants in both conditions once a month after the end of the program to maintain contact and interest in completing post-program evaluations.”
5-xii) No co-intervention was provided.

6a) Only primary outcomes were applicable. “The eligible participants then filled in the pre- post-, and follow-up assessments online”. The primary outcomes are indicators of physical and mental well-being, which were mental well-being, psychological distress, life satisfaction, energy, sleep disturbance, and pain.
6a-i) Online questionnaires were validated before release to ensure question items were presented correctly and data were accurately captured in the backend database. Reliable and valid measures were used and was described in the “measures” section
6a-ii) Yes. “Participants were instructed to call and/or email our research assistant for clarification in case of questions or problems during the course of the intervention”. They can also email or call the research team on feedback and queries.
6b) No change to trial outcomes after the trial commenced.

7a-i) Assuming a small to medium effect size, 60 participants per group will be needed to achieve 80% power. Based on previous studies on Internet interventions, we expected a 70% attrition rate at follow-up. Therefore, a sample size of 400 participants at pre-program would be enough. We did not cut the recruitment after the expected sample size was reached and it was open to the public during the study period.
7b) Not applicable.

8a) Yes. “Participants received an activation link via email and were then randomly assigned to one of the two conditions by computer-generated numbers.”
8b) True randomization was used

9) Randomization was performed automatically by the website programming logic after participants gave consent to participating in the study. No one had prior knowledge regarding which condition a participant got assigned to till the assignment was completed.

10) Random allocation was performed programmatically by the web program implementation.

11a) The study was an open-label trial and participants were not blinded to their assigned condition
11a-i) The study was an open-label trial and participants were not blinded to their assigned condition. To respond and answer participants' queries, supporters were not blinded to the condition as well.
11a-ii) Participants were told that they would be assigned to either one of the two conditions that can help to improve mental health. Since both the Internet-based cognitive-behavioral training and the Internet-based mindfulness-based training aim to improve well-being, it is unlikely that participants would know which intervention was the "comparator".
11b) Both conditions "consisted of eight 30- to 45-minute sessions." "At the end of each session, participants were provided with homework assignments to practice what was learned and apply the skills in their daily lives" for both conditions.

12a) "All analyses were conducted using SPSS 20.0. Linear mixed models were conducted to test if both conditions showed improvements in all outcomes over time. Compound symmetry covariance was used and missing data were treated using restricted maximum likelihood estimation. Model for each outcome variable consisted of the time effect, group effect, and the interaction effect of time by group. When the main effect of time was significant, follow-up tests were conducted to compare the outcomes in post-program and follow-up program with the pre-program and results were adjusted with Bonferroni correction"
12a-i) Yes. "Missing data were treated using restricted maximum likelihood estimation"
12b) "When the main effect of time was significant, follow-up tests were conducted to compare the outcomes in post-program and follow-up program with the pre-program and results were adjusted with Bonferroni correction."

X26) Yes. "Clinical ethics approval was obtained from the principal investigator's institution (Joint Chinese University of Hong Kong – New Territories East Cluster Clinical Research Ethics Committee) as well as from the Hospital Authority Kowloon Central/East Cluster and the Department of Health of Hong Kong."
x26-ii) Yes. "Eligible individuals were given detailed information about the study aims, length of the program, participant involvement, and the assignment of intervention through randomization. They were also informed that the study was conducted by the Department of Psychology at The Chinese University of Hong

Kong. Participants provided informed consent by clicking the “I agree” button at the bottom of the study description page.”
X26-iii) “When a participant fell below a score of 13 or answered 0 or 1 on any of the items on the Well-Being Index, first tier supporters would refer them to second tier supporters (who were clinical psychologists) to evaluate their mental health status, address their questions, and/or make referrals for more intensive treatments as needed. Each participant was monitored through weekly self-report measures as well as their first and/or second tier supporters.”

13a) Yes. Figure 1 showed all the details.
13b-i) Yes. Figure 1 one showed the attrition over the study

14a) Yes. “Participants were recruited between July 2013 and March 2015”
14a-i) No critical “secular events” fell into the study period.
14b) The trial was ended because the funding has ended.

15) The concept of care providers and centers in each group was not applicable in the current study. “Table 1 and Table 2 display the baseline characteristics of the participants in both conditions.”
15-i) Yes. “Table 1 and Table 2 display the baseline characteristics of the participants in both conditions. Overall, participants had a mean age of 32.62 years ($SD = 12.54$), were predominantly female (74.3%), with half of them being college graduates (52.5%). About one-thirds (34.6%) were college students and about half (51.1%) were working full-time (see Table 1). Both groups reported similar treatment expectancy and credibility (ts < .57, ps > .30). Findings showed that both groups expressed similar CSQ usage satisfaction, $t(211) = -.07$, $p = .94$.”

16) Yes. “. Our sample consisted of those who, after randomization, completed the pre-survey and received course materials ($N = 1{,}255$). About one-fifth of the participants (n=253) completed the entire 8-session program, 17.0% ($n = 213$) completed the post-program survey, and 10.1% ($n = 127$) completed the 3-month follow-up.”
16-ii) All analyses were conducted using intent-to-treat and “missing data were treated using restricted maximum likelihood estimation”.

17a) All relevant results were reported in the result section and in Table 3 and Table 4.

17a-i) “Usage is defined as the time (in minutes) spent in the previous week on browsing the website and practicing the assigned homework. Participants reported these figures at the beginning of every session.” “In terms of utilisation, iMIND group (M = 189.89, SD = 501.00) spent more time browsing the course content than their iCBT counterparts (M =135.98, SD = 347.08), t(1063.9) = -2.20, p < .05). However, iCBT group (M = 240.63 minutes, SD = 578.52) spent more time on homework assignment than iMIND group (M = 118.42, SD = 401.42) , t (1162.3) = 4.37, p < . 001.”
17b) Binary outcomes are not applicable in the present study.

18) “When the main effect of time was significant, follow-up tests were conducted to compare the outcomes in post-program and follow-up program with the pre-program and results were adjusted with Bonferroni correction.”
18-i) Not applicable.

19) No harmful effect was exerted.
19-i) No privacy breaches or technical problems happened during the course of the study.
19-ii) There was no qualitative feedback gathered in the study.

22)Yes. “The present study developed and evaluated the effectiveness of the Internet-based mindfulness training in comparison with an Internet-based cognitive-behavioral training on college students and young working adults in Hong Kong. Results showed that the Internet based mindfulness training was as effective as the widely-supported Internet cognitive-behavioral training in improving mental well-being, psychological distress, life satisfaction, energy level, and sleep disturbance at the end of the 8-week program. Furthermore, users’ perceived credibility, expectancy, and satisfaction of both programs were similar.”

22-ii) Yes. “Although improvements in outcomes were observed at post-program, the improvements for psychological distress were not maintained at 3-month follow-up. This could be the result of reduced practice or application of skills learnt on the websites. In addition, no significant improvement in pain was shown at post-program and 3-month follow-up. This might due to the low level of pain observed within this group of population. The floor effect might have limited the possibility in detecting improvement in pain at post-program and 3-month follow-up. The two Internet-based interventions in this study yielded similar results. Future studies can explore how

individual differences may affect intervention benefits. It may be possible that cognitive styles can play a role in the receptivity of iMIND and iCBT and matching their styles with the treatment approach may maximize the outcome."

20-i) Yes. "The present study has several limitations. First, our target population was college students and young working adults. By nature, our sample is skewed toward those who were educated or were employed. As our programs were Internet-based, it is possible that they appealed to a selective group in the population who were more comfortable in accessing interventions over the Internet with their personal computers. They might have higher mental health literacy and be more willing to participate in Internet-based mental health programs. These biases in our sample limit the generalizability of our findings to all segments of the population (e.g., less educated individuals, older adults). It is possible that the delivery of mental health materials over the Internet may only be appropriate for specific segments of the populations, rather than the entire population. Future studies should focus on how Internet-based interventions can cater to different segments of the populations through various adaptations. Second, the attrition rate of our study is high. High attrition rate has been a perennial problem for Internet-based interventions. Similarly high attrition rates have been reported in other Internet-based mental health programs. For example, Christensen and colleagues [54] reported an attrition rate of 74% in its Internet-based CBT program for depression. Another study reported an attrition rate of 98.8% in an Internet-based CBT for panic disorder [55]. A study based on the MoodGym had an attrition rate of 73.9% for trial participants and 99.2% for public registrants [56]. In a more recent systematic review of Internet-based interventions for anxiety and depression, the completion of protocol rates for depression sites ranged from 43% to 99% [57]. Moreover, during the inception of our project, smartphones and tablets were not as omnipresent as they are today. Because of that, our online modules were designed using Adobe Flash and thus only catered for desktop viewing, which may deter usage. Third, we did not include a waitlist control group in this study. As this study aimed to compare Internet-based mindfulness training with a well-supported Internet-based cognitive behavioral training, and previous study has found Internet-based mindfulness training to have significant improvements in mental well-being than waitlist control [26] we decided not to have a waitlist control in the present study so not to withhold intervention from our participants. Fourth, we did not ask whether our participants received any other psychological intervention during the study period. Thus, the findings may potentially be attributed to additional intervention that the participants have received. Finally, the present study found that those who quit the programs scored lower on mental well-being measures, energy level, mindful

awareness, and treatment expectancy at the outset. To ensure interventions are catered to those most in need, future studies should explore the reasons behind attrition and identify corresponding remedies. As suggested by existing research, utilization can potentially be promoted via built-in incentives, personalized feedback, and user collaboration [58].”

21-i) Generalizability to other populations
Yes. “Our target population was college students and young working adults. By nature, our sample is skewed toward those who were educated or were employed. As our programs were Internet-based, it is possible that they appealed to a selective group in the population who were more comfortable in accessing interventions over the Internet with their personal computers. They might have higher mental health literacy and be more willing to participate in Internet-based mental health programs. These biases in our sample limit the generalizability of our findings to all segments of the population (e.g., less educated individuals, older adults). It is possible that the delivery of mental health materials over the Internet may only be appropriate for specific segments of the populations, rather than the entire population. Future studies should focus on how Internet-based interventions can cater to different segments of the populations through various adaptations.”
21-ii) There would be no difference

23) “Trial Registration: Chinese Clinical Trial Registry (ChiCTR) ChiCTR-TRC-12002623”

24) The protocol in held within the research team and is available upon request.

25) Yes. “We would like to acknowledge the Health and Health Services Research Fund (Ref. No. 09100711) for funding this project.”

X27-i) Yes. “The study was supported by the Health and Health Services Research Fund (Ref. No. 09100711). The first author of the study, Winnie Mak, is one of the developers of the content of the trials but do not own the source code of the website.”
